# The Demethylase Activity of FTO (Fat Mass and Obesity Associated Protein) Is Required for Preadipocyte Differentiation

**DOI:** 10.1371/journal.pone.0133788

**Published:** 2015-07-28

**Authors:** Meizi Zhang, Ying Zhang, Jun Ma, Feima Guo, Qian Cao, Yu Zhang, Bin Zhou, Jijie Chai, Wenqing Zhao, Renbin Zhao

**Affiliations:** 1 Space Biology Research and Technology Center, China Academy of Space Technology, Beijing Engineering Research Center of Space Biology, Beijing, 100190, China; 2 School of Life Sciences, Tsinghua University, Beijing, 100084, China; Hosptial Infantil Universitario Niño Jesús, CIBEROBN, SPAIN

## Abstract

FTO (fat mass and obesity associated gene) was genetically identified to be associated with body mass index (BMI), presumably through functional regulation of energy homeostasis. However, the cellular and molecular mechanisms by which FTO functions remain largely unknown. Using 3T3-L1 preadipocyte as a model to study the role of FTO in adipogenesis, we demonstrated that FTO is functionally required for 3T3-L1 differentiation. FTO knock-down with siRNA inhibited preadipocyte differentiation, whereas ectopic over-expression of FTO enhanced the process. The demethylase activity of FTO is required for differentiation. Level of N^6^-methyladenosine (m^6^A) is decreased in cells over-expressing FTO. In contrast, overexpression of R96Q, a FTO missense mutant lack of demethylase activity, had no effect on cellular m^6^A level and impeded differentiation. Treatment with Rosiglitazone, a PPARγ agonist, could overcome the differentiation inhibition imposed by R96Q mutant, suggesting the effect of FTO is mediated through PPARγ.

## Introduction

Since the original publication of association between genetic variation in FTO and body mass index [1–4], considerable efforts have been dedicated to elucidating the molecular mechanism of FTO in modulating energy homeostasis. Studies of genetically engineered mouse models have highlighted the level of complexity in uncovering the role of FTO in regulating body composition and energy metabolism[5]. Global germ line KO of FTO resulted in reduced body weight and growth retardation [6–8]. While most germ line inactivation or over-expression models support a positive correlation between FTO activity and fat mass[7–9], adult onset loss of FTO resulted in increased fat mass and reduced lean mass [10]. In addition, knock-down of FTO activity in a sub-region of hypothalamus only led to mild phenotypes comparing to global inactivation, suggesting FTO exerts function in sites beyond hypothalamus[10].

FTO belongs to the Fe (II) and oxoglutarate-dependent AlkB oxygenase family, and was originally shown to catalyze the oxidative demethylation of 3-methylthymidine (m^3^T) or 3-methyluracil (m^3^U) in single strand DNA/RNA [11–14]. In 2011 Jia et al reported that FTO could demethylateN^6^-methyladenosine (m^6^A) in RNA, and exhibited much higher activity to m^6^A versus m^3^U *in vitro*[15]. The authors also demonstrated that over-expression of FTO led to a decrease of m^6^A level in cultured cells, suggesting that m^6^A is a physiological substrate of FTO. Transcriptome-wide studies with m^6^A-specificRNA immune-precipitation and next generation sequencing revealed m^6^A modifications are widespread, dynamically and tissue-specifically regulated [16, 17]. Analysis of m^6^A mRNA in tissues deficient of FTO led to identification of potential transcripts as the targets of demethylation, and established a link between the demethylase activity and physiological processes regulated by FTO [18].

FTO is ubiquitously expressed, with highest levels in brain and hypothalamus [1, 2, 11]. Although much attention has been devoted to FTO function in brain/hypothalamus [8, 11, 19–24], studies beyond the central nervous system are emerging [25, 26]. Adipose tissue is the primary site for lipid storage, and acts as an endocrine organ regulating energy status via secreting and responding to hormones[27]. Several studies reported the effect of FTO over-expression or deficiency on gene expression changes in adipose tissue [7, 28], and progresses have been made towards uncovering the role of FTO in adipogenesis and energy expenditure [29–31]. In this study, we used murine 3T3-L1 preadipocyte as a model[32] and generated cell lines stably expressing wild-type FTO or a mutant lack of demethylase activity. With these tools and global gene expression profiling, we characterized that the demethylase activity of FTO is required for adipogenesis and discuss the pathways that are possibly involved.

## Materials and Methods

### Cell Culture and Adipocyte Differentiation

3T3-L1 cells were purchased from American Type Culture Collection (ATCC). Cells were cultured in high glucose DMEM (Gibco, cat.11995-065) supplemented with 10% bovine calf serum (Hyclone, cat.SH30118.02), 100 U/ml penicillin and 100 mg/ml streptomycin (Gibco, cat.15140-122) in a 5% CO_2_ humidified atmosphere. For 3T3-L1 differentiation, post-confluent preadipocytes were incubated with a cocktail of insulin (1 μg/ml, Sigma, cat.I5500), dexamethasone (1 μM, Sigma, cat.D4902), and 3-isobutyl-1-methylxanthine (0.5 μM, Sigma, cat.I7018) in DMEM supplemented with 10% fetal bovine serum (Hyclone) for 48 hours, followed by culture with DMEM, 10% fetal bovine serum and 1 μg/ml insulin for another 48 hours. The media were then removed and replaced with DMEM plus 10% fetal bovine serum until collection for differentiation assessment. For PPARγ pathway studies, rosiglitazone (10 μM, Sigma, cat.R2408) was added to the media at day 0 of induction and throughout the differentiation process.

Oil Red-O (Sigma, cat.O0625) staining was performed on day 6 of differentiation following manufacturer’s instruction. In brief, cells were washed twice with PBS and fixed with 10% formalin in PBS for 15 min. After two washes with PBS, cells were stained for at least 1 hour in freshly diluted Oil Red-O solution (stock solution: 0.5% Oil Red-O in isopropanol; for dilution, water:stock solution is 4:6). The staining solution was then removed and cells were washed 3 times with PBS before imaging. To quantify lipid staining, Oil Red-O was extracted by adding 100% isopropanol after imaging, and the absorbance of the extract was measured at 540 nm.

### FTO Knockdown with siRNAs

All siRNAs were purchased from Invitrogen (siFTO-1:UUAAGGUCCACUUCAUCAUCGCAGG, siFTO-2:CAGGCACCUUGGAUUAUAUTT). Cells were plated at 3x10^5^ per well in 6-well plates, and grown for 24 hours in normal growth media. For knock-down experiments, cells were rinsed with Opti-MEM (Invitrogen, cat.31985-062) and incubated with 100 nM siRNA and Lipofectamine2000 (Invitrogen, cat.13778075) for another 24 hours. The transfection media were then replaced with DMEM supplemented with fetal bovine serum, and cells were ready for subsequent differentiation induction.

### RNA Isolation and RT-qPCR

Total cellular RNA was isolated from 3T3-L1 cells using RNeasy Protect Mini Kit (Qiagen, cat.74104). First-strand cDNA synthesis was performed using oligodT-primers with SuperScript III reverse transcriptase (Invitrogen, cat.18080-044). The expression level of each gene was determined with ABI 7500HT Sequence Detection System (Applied Biosystems). Reactions were performed in triplicates with 12.5 μl of Power SYBR Green Master Mix, 300 nM primers, 20 ng of cDNA template or control/nuclease-free water in a final volume of 25 μl. The RT-qPCR reaction consisted of an initial denaturation for 10 min at 95°C, followed by 40 cycles of 95°C for 15 sec, 60°C for 60 sec. Threshold cycle (Ct) values were calculated using ABI SDS 3000 software (Applied Biosystems). All the gene expression data during 3T3-L1 differentiation were normalized using Ywhaz. Primer sequences:

Fto:TTCATGCTGGATGACCTCAATG/GCCAACTGACAGCGTTCTAAG

Ywhaz:GAAAAGTCTTGATCCCCAATGC/TGTGACTGTCCCAATTCCTT

Actb: GCTCGTCGACAACGGCTC/ CAAACATGATCTGGGTCATCTTCTC

Cd36:GATGTGGAACCCATAACTGGATTCAC/GGTCCCAGTCTCATTTAGCCACAGTA

aP-2: ACATACAGGGTCTGGTG/CAGCACTCACCCACTTCTTTCAT

Plin1: TGCTGGATGGAGACCTC/ACCGGCTCCATGCTCCA

Adipoq:ATGCCGAAGATGACGTTACTACA/CCTGCACAAGTTCCCTTGGG

Fabp5:TGAAAGAGCTAGGAGTAGGACTG/CTCTCGGTTTTGACCGTGATG

Pparg:TCGCTGATGCACTGCCTATG/GAGAGGTCCACAGAGCTGATT

### FTO mutant constructs and cell lines

The cDNA of wild-type FTO was amplified by PCR from FTO expressing plasmid pcDNA3.1-N-6His-3myc-FTO-1-505 [14]. Primers bearing Hind III and Xho I sites were used to generate PCR fragments which were subcloned into pcDNA3.1 mammalian expression vectors (Invitrogen). The R96Q mutant of FTO was described previously [14].The cDNA of R96Q was amplified by PCR and cloned into pcDNA3.1 mammalian expression vectors. 3T3-L1 cells were plated at 3x10^5^ per well in six-well plates and transfected with constructs using X-tremeGENE HP DNA(Roche,cat.6366236001) following manufacturer’s instruction. Transfection media were removed after 24 hours and cells were cultured in normal growing media for 48 hours. Cells were then passed 1:10 into 10 cm dishes and grown in selective media with G418 (600 μg/ml, Sigma, cat.A1720) for 21 days when single colonies were picked from each culture.

### Western-blot analysis

Cells were lysed in RIPA buffer with a cocktail of protease inhibitors (Sigma, cat.P8340). 20 μg of protein extracts were separated by SDS-PAGE and transferred to PVDF membranes (Millipore, cat.GVWP2932A). Membranes were blocked with 5% nonfat milk, followed by overnight incubation with primary antibodies against FTO (1:500, Abcam, cat.ab92821), β-actin (1:1000, Cell Signaling Technology, cat.#3700), or Myc (1:500, Abcam, cat.ab9106). Detection was made with HRP conjugated secondary antibody (1:5000, Santa Cruz) and enhanced with chemo-luminescence detection system (Millipore, cat.WBKLSO100).

### Immunofluorescent Cell Staining

Cells growing on coverslips were fixed with 4% paraformaldehyde, permeabilized with 0.01% Triton X-100 (Sigma,cat.9002-93-1), blocked with PBST-0.5% BSA, and incubated sequentially with anti-FTO monoclonal antibody (1:100; Abcam) and FITC-conjugated secondary antibody (1:2000, ZSGB-BIO, cat.ZF-0312). All cells were counterstained with hoechst33342 and mounted in anti-quenching medium (Solarbio, cat.S2100). Imaging analysis was carried out with Nikon A1Rsi confocal microscope.

### Analysis ofm6A levels in mRNA using dot blot

Total RNAs were isolated from cells 24hr after adipogenic differentiation with TRIZOL reagent (Invitrogen, cat.15596-018). Cellular mRNAs were isolated with Poly(A) Purist Kit (Ambion, cat.AM1916), followed by rRNA removal using RiboMinus Transcriptom Isolation Kit (Invitrogen, cat.K1550-01). The concentration and quality of mRNA were determined by NanoDrop (Thermo) and Agilent 2100 bioanalyzer. Purified mRNA was denatured at 95°C for 5 min and then chilled on ice. Two fold serial dilutions were spotted onto Amersham Hybond-N+ membranes (GE Healthcare, cat.RPN303B). After crosslinking at 80°C for 2 hours, the membranes were blocked with 5% nonfat milk in TBST for 1 hour, and incubated with rabbit anti-m^6^A antibody (1:2000, SySy, cat.202003) overnight at 4°C. Membranes were then incubated with peroxidase-conjugated AffiniPure goat anti-rabbit IgG (H+L) for 1 hour at room temperature, and visualized by Immobilion western Chemoluminescent HRP Substrate (Millipore,cat.WBKL S0050). The intensity of each spot was quantified using ImageJ software (NIH, USA).

### Gene expression profiling

Gene expression profiling was performed with AffymetrixGeneChip Mouse Gene 1.0 ST arrays. After scanning, CEL files were processed at gene level using RMA algorithm of the Affymetrix Expression Console Software 1.1. Probesets without RefSeq annotations were removed from further analysis. Differentially regulated genes were identified using the univariate test in BRB-ArrayTools with a P value of 0.01 as the significance threshold[33]. Pathway enrichment analysis was applied to significantly regulated genes using the Database for Annotation, Visualization and Integrated Discovery (DAVID)[34]. Significantly enriched pathways were identified using a P value of 0.05 as the threshold. The microarray data have been submitted to NCBI GEO database, accession number GSE69313.

## Results

### FTO knock-down inhibits 3T3-L1 differentiation

**Fig 1:**
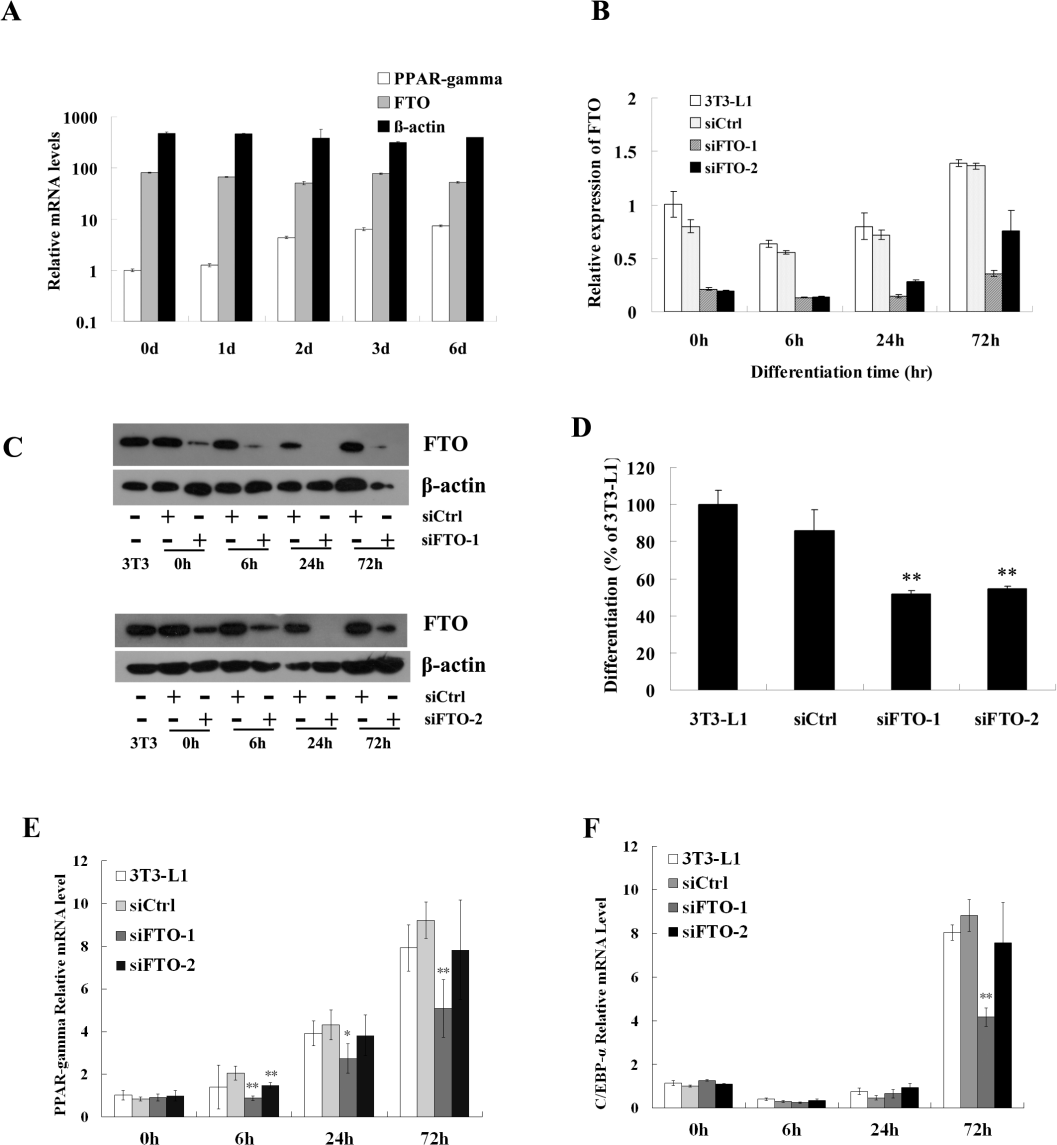
FTO knockdown inhibits differentiation of 3T3-L1 preadipocyte. (A) The mRNA levels of FTO, PPAR-γ, and β-actin at different time point during3T3-L1 differentiation was analyzed with RT-qPCR. The relative mRNA levels were determined by the ratio to that of PPAR-γ at time 0.The level of FTO mRNA (B) and protein (C) in cells treated with siRNAs and controls were assessed with RT-qPCR and Western Blot. (D) 3T3-L1 preadipocyte differentiation was inhibited by FTO knock-down. The extent of differentiation was assessed by Oil Red-O staining of intracellular triglyceride and measuring the extract’s absorbance at 540 nm. (E) Expression levels of PPAR-γ in 3T3-L1 cells and those transfected with control or FTO siRNAs at different time point during differentiation. (F) Expression levels of C/EBP-α in 3T3-L1 cells and those transfected with control or FTO siRNAs at different time point during differentiation. All gene expression data are represented as mean ± SD of triplicates.* stands for p<0.05 and ** stands for p<0.01 in Student’s t-test (siFTO vs. siCtrl).

We first examined FTO expression during 3T3-L1 differentiation. Cells were treated with adipogenic cocktail MDI (see materials and methods) and RNA was collected right before induction and at 1, 2, 3 and 6 days post induction. Quantitative RT-PCR (RT-qPCR) analysis showed that FTO is expressed at relatively high level over 6 days of differentiation (Fig 1A). In contrast, the level of PPAR-γ the master regulator of adipogenesis, is low in preadipocyte but dramatically increased during adipocyte maturation.

Next, we investigated the effect of FTO knock-down on 3T3-L1 differentiation. Cells were transiently transfected with two siRNAs, siFTO-1 or siFTO-2, and treated with MDI 24 hours after transfection. The FTO RNA level was significantly reduced compared to control, and only started to bounce back 72 hours post induction (Fig 1B). Western-blot confirmed the protein level of FTO was suppressed (Fig 1C). The extent of differentiation was assessed with Oil Red-O staining of intracellular triglyceride and quantified by extraction and measurement of absorbance at 540 nm. As shown in Fig 1D, both siRNAs caused about 50% reduction of staining versus control, suggesting FTO function is required during adipocyte differentiation. To confirm the Oil Red-O staining results, mRNA levels of PPARγ and C/EBPα, two master regulators of adipogenesis, were also measured. As shown in Fig 1E and 1F, siFTO-1 reduced the expression of both PPARγ and C/EBPα. The effect of siFTO-2, although not as potent as siFTO-1, demonstrated similar trend. These observations confirmed FTO is required for adipocyte differentiation.

### The demethylase activity of FTO is required for 3T3-L1 differentiation

To confirm the role of FTO in preadipocyte differentiation and investigate whether the demethylase activity is required, we decided to generate 3T3-L1 cell lines stably over-express wild-type (wt) FTO or a mutant lack of demethylase activity (R96Q). The Arg96 in FTO is critical for substrate binding, and mutation of Arg to Gln (R96Q) abolished the demethylase activity *in vitro*[14].

**Fig 2:**
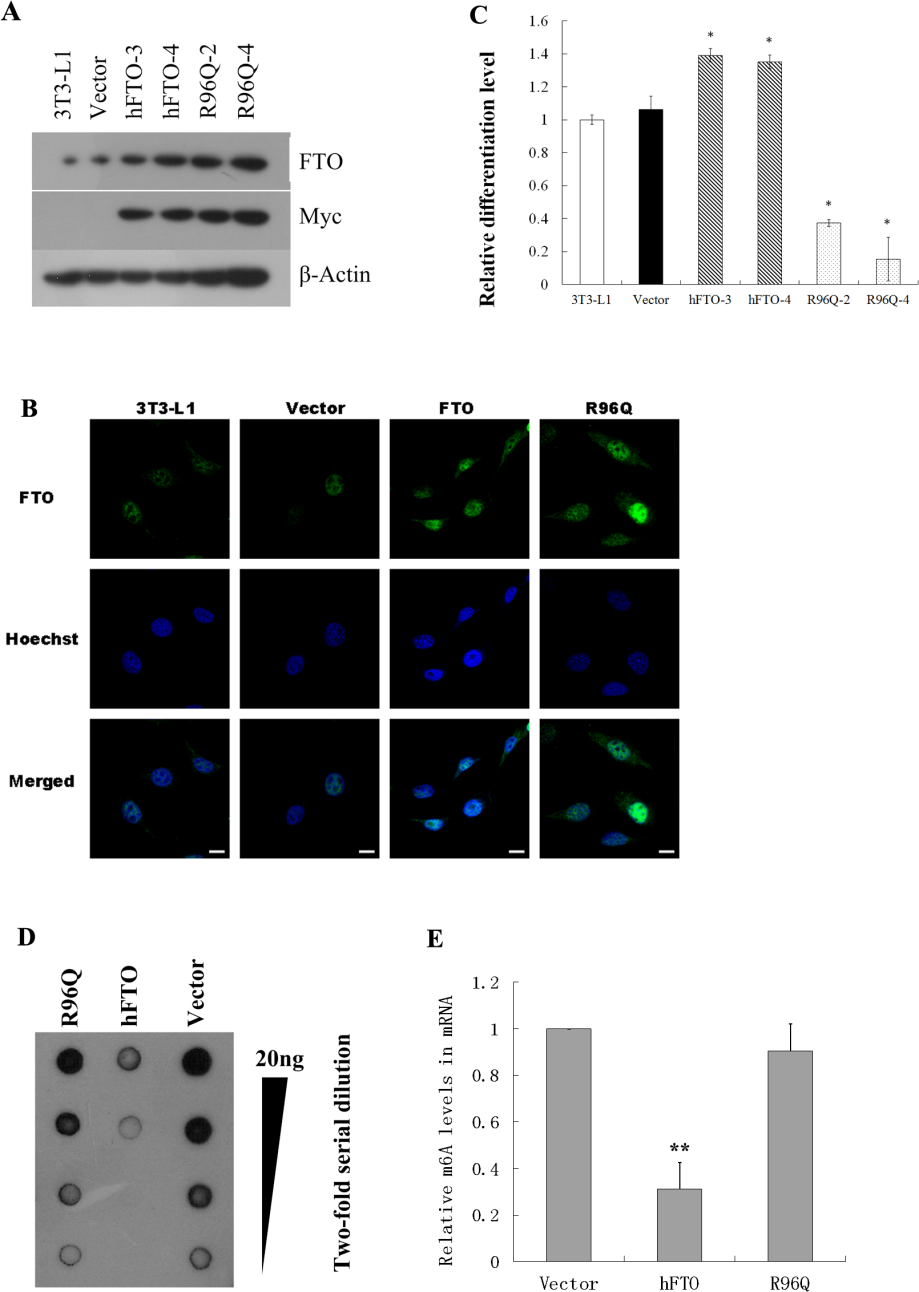
The demethylase activity of FTO is required during 3T3-L1 differentiation. Two lines of 3T3-L1 stably expressing wt FTO (hFTO-3 and hFTO-4) or R96Q (R96Q-2 and R96Q-4) were generated. (A) The protein expression level was confirmed by Western-blot using FTO and Myc antibodies, with β-actin as a loading control. (B) The sub-cellular localization of hFTO-4 or R96Q-4 was determined with confocal fluorescence imaging. Scale bars of 10μm are shown in the bottom row of images. (C) 3T3-L1differentiation was enhanced by over-expression of wt FTO and inhibited by R96Q. Level of differentiation was measured by extent of Oil Red-O staining at day 6 post differentiation induction (see materials and methods). Demethylase activities of wt hFTO-4 and R96Q-4 in 3T3-L1 cells were determined by dot blotting (D) and quantified by Grayscale analysis with ImageJ software (E). Data are represented as means± SD of four replicates. * stands for p<0.05 and** stands for p<0.01in Student’s t-test.

Myc-tagged constructs were generated to ectopically express wtFTO as well as the R96Q mutant in 3T3-L1 cells. Independent clones of corresponding stable cell lines were selected based on PCR screening and subsequent sequencing. All clones showed comparable growth rate (data not shown). The expression of ectopic wt and mutant FTO proteins were verified with Western-blot, using antibodies to FTO or Myc (Fig 2A). In agreement with previous report [11], both FTO and R96Q are primarily located in the nucleus (Fig 2B). Consistent with the siRNA knock-down results, over-expression of FTO led to enhanced differentiation compared to cells transfected with vector alone (Fig 2C). Unexpectedly, ectopic expression of the demethylase-deficient mutant R96Q led to drastic inhibition of differentiation to a level that is only 15–40% of controls (Fig 2C). The dominant negative effect of R96Q on 3T3-L1 differentiation was observed with two independent clones designated R96Q-2 and R96Q-4, arguing against the possibility of integration site peculiarity.

To examine the impact of ectopic expression of FTO or R96Q on cellular m^6^A level, total mRNAs were purified from cell lines stably expressing either wt hFTO-4or R96Q-4 24hr after adipogenic differentiation. Level of m^6^A was determined by dot blotting with antibody specifically recognizing N^6^-methyladenosine [16]. Over-expression of wt FTO in 3T3-L1 cells resulted in significant reduction of m^6^A level compared to vector transfected cells, whereas the demethylase-deficient mutant R96Q had minimum effect on m^6^A level (Fig 2D and 2E).These results suggest the N^6^-methyladenosine demethylase activity of FTO is required during adipocyte maturation.

### PPAR-γ agonist could rescue the differentiation inhibition imposed by R96Q

**Fig 3:**
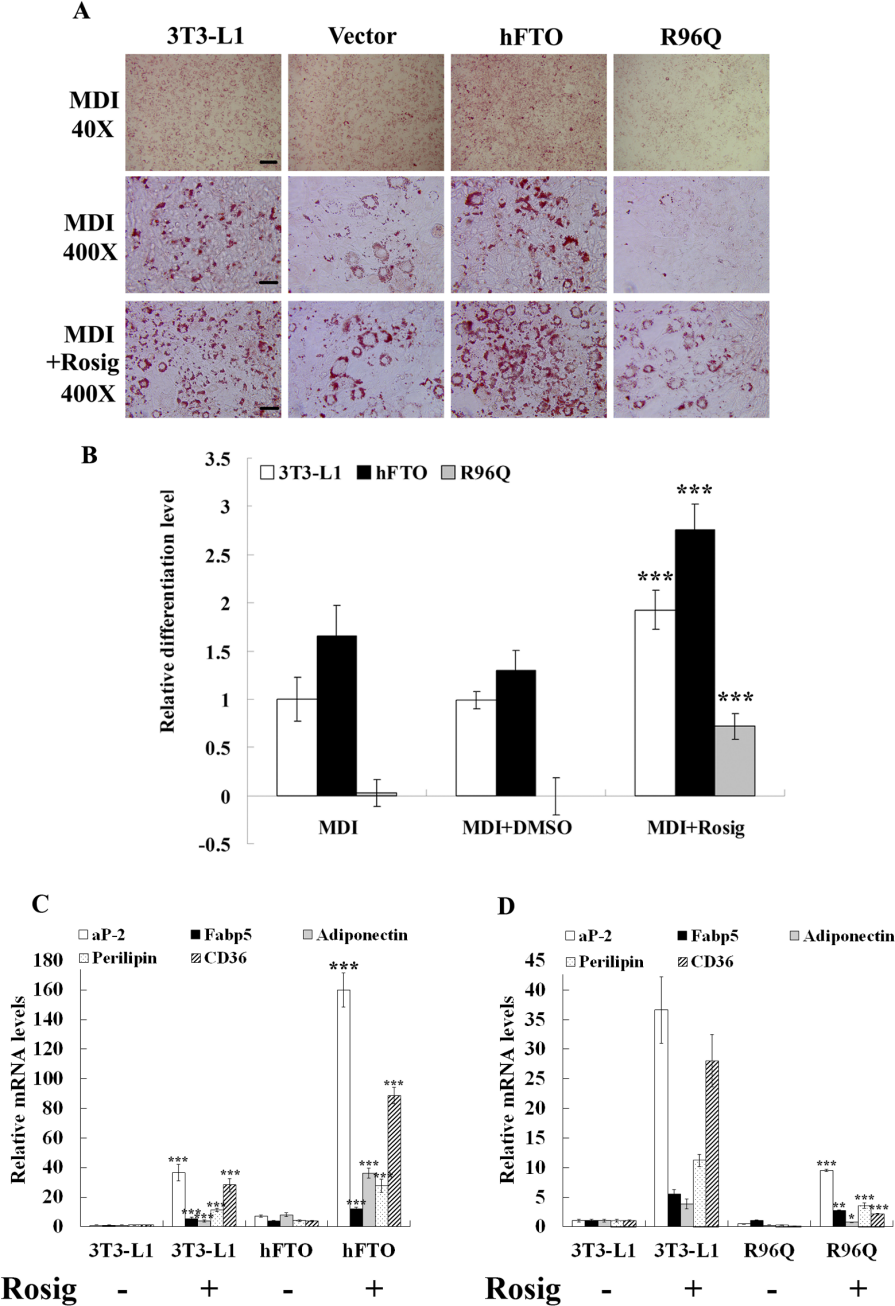
Rosiglitazone rescued adipogenesis inhibition in 3T3-L1 cells expressing R96Q. (A) Oil Red-O staining of 3T3-L1 cell lines treated with MDI (top and middle row), or MDI plus Rosiglitazone (bottom row). Scale bars are 500μm in 40x images and 50μm in 400x images respectively (left column). (B) Level of differentiation in 3T3-L1 lines expressing wt hFTO-4 or R96Q-4, measured by the extent of Oil Red-O staining. Expression levels of five PPARγ target genes (aP-2, Fabp5, Adiponectin, Perilipin and CD36) were evaluated in 3T3-L1 over expressing wt hFTO-4 (C) or R96Q-4(D) at day 3 post differentiation induction. All gene expression data are represented as mean ± SD of triplicates.* stands for p<0.05, ** stands for p<0.01 and *** stands for p<0.001 in Student’s t-test (Rosig versus DMSO)

PPARγ is a member of the nuclear hormone receptor superfamily, and activate the transcription of many genes characteristic of adipocyte features. PPARγ has been shown to be both necessary and sufficient for adipogenesis, and thus considered the master regulator of this process [32, 35]. To evaluate whether the effect of FTO is mediated through PPARγ, we added Rosiglitazone, a PPARγ agonist, to the differentiation cocktail MDI (see materials and methods).At day 6 post induction, the differentiation extent was significantly increased by Rosiglitazone in cell lines overexpressing wt hFTO-4, R96Q-4 or controls, as measured by Oil Red-O staining (Fig 3A). The effect of PPARγ agonist is particularly remarkable in R96Q-4 expressing cells, where the level of differentiation was restored to over 30% of controls, versus to less than 3% in MDI treatment only (Fig 3B).We also measured the expression levels of several adipocyte markers such as aP-2, Perilipin, Fabp5, Cd36 and Adiponectin, all showed increased expression by Rosiglitazone treatment (Fig 3C and 3D). In addition, the differentiation suppression imposed by R96Q-4 was also partially restored by Rosiglitazone (Fig 3D). These results suggest that FTO exerts its effect upstream of PPARγ during adipogenesis.

### Microarray analysis of gene expression changes caused by FTO inhibition

To elucidate the mechanism of FTO-mediated regulation of adipogensis, we profiled gene expression patterns using microarray. Normal, control (random siRNA), siFTO-1, or siFTO-2 transfected 3T3-L1 cells (three replicates of each) were induced to differentiation with MDI, and RNA samples were collected at 0, 6, 24 and 72 hours post induction. Genes with significant expression changes were identified by comparing siFTO transfected cells to those treated with random siRNA (see materials and methods). Overall, siFTO-1 and -2 induced similar gene expression changes at different time point, although the effect of siFTO-1 is more potent. Thus we used the significant genes from siFTO-1 for further pathway analysis. A total of 970 differentially regulated genes were identified, among which 567 at 6 hr, 309 at 24 hr, and 94 at 72hr, respectively (S1 Table).

**Fig 4:**
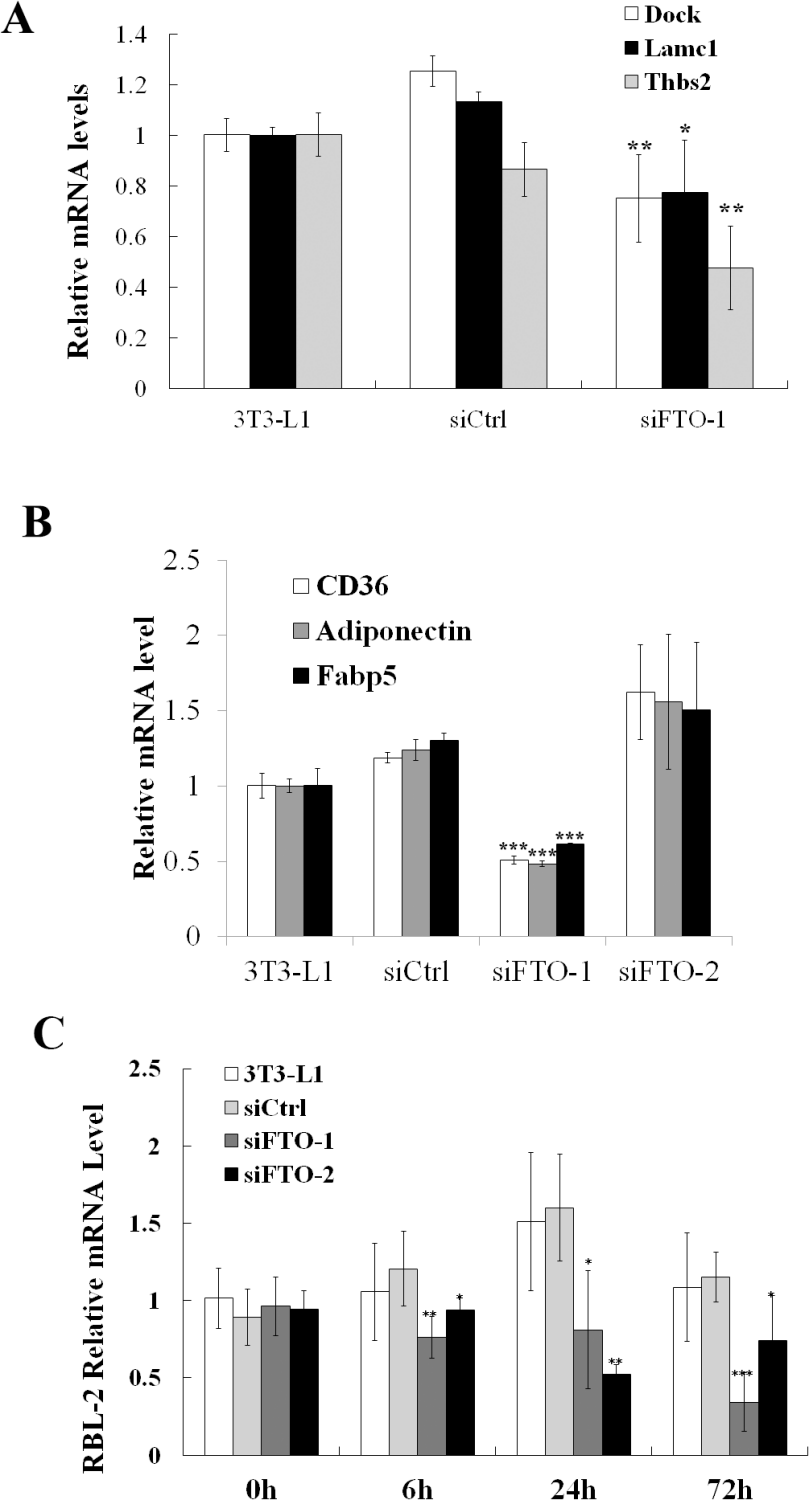
Confirmation of microarray findings with RT-qPCR. (A) Three genes from the focal adhesion, cytoskeleton and ECM gene set (Lamc1, Dock1 and Thbs2) were selected and confirmed to be decreased 6hr following adipogenic induction in FTO knock-down cells. (B) Three PPARγ target genes (CD36, Fabp5 and Adiponectin) were selected and verified to be down-regulated 3 days following adipogenic induction in siFTO transfected cells. All gene expression data are represented as mean ± SD of triplicates. * stands for p<0.05, ** stands for p<0.01 and *** stands for p<0.001in Student’s t-test (siFTO versus siCtrl).

Most genes differentially regulated by FTO knockdown exhibited moderate changes of less than 2-fold. It is generally believed that multiple genes in a particular pathway are co-regulated to accomplish physiologically relevant function, despite relatively small changes in individual genes[36]. To identify pathways that are modulated by FTO knock-down, pathway enrichment analysis was performed using DAVID[34]. At 6 hr, several genes in the Wnt signaling pathway were significantly up-regulated, including Wnt10b, which was previously reported to inhibit adipogenesis [37, 38]. In contrast, a number of genes in focal adhesion, actin cytoskeleton and ECM receptor interaction were down-regulated 6hr post induction (Table 1), which probably reflected the changes in cell shape and interaction with extracellular matrix during differentiation [39–42]. At 72hr, many PPAR-γ target genes [43, 44] were down-regulated (Table 1), again consistent with our phenotypic observation of reduced adipogenesis with Oil Red-O staining. Some of the expression changes detected by microarray were confirmed with RT-qPCR (Fig 4).

**Table 1:** Pathways significantly impacted by siRNA knock-down of FTO in 3T3-L1 cells at 6 hr and 72 hr post differentiation induction.

Enriched Pathways	Symbol	p value	Fold change (siFTO/siCtrl)	m6A sites
WNT signaling pathway (6hr)	Apc2	5.10E-03	1.49	+
	Wnt10b	9.35E-03	1.47	+
	Wnt8b	8.15E-03	1.33	+
	Wnt2	9.97E-03	1.44	-
	Wnt7a	5.84E-03	1.36	+
Focal adhesion pathway/Regulation of actin cytoskeleton/ECM-receptor interaction (6hr)	Dock1	5.22E-03	0.72	+
	Flnb	5.83E-03	0.7	+
	Gna13	5.78E-03	0.72	+
	Igf1r	3.19E-03	0.69	+
	Itga1	4.43E-03	0.72	-
	Itga6	3.62E-04	0.55	+
	Lama4	8.28E-04	0.67	-
	Lamc1	1.87E-03	0.67	+
	Mapk3	8.94E-03	0.76	-
	Mapk9	8.22E-03	0.77	+
	Mylk	1.80E-03	0.67	-
	Pak3	5.81E-03	0.77	+
	Parvb	1.30E-03	0.66	+
	Ssh2	1.18E-03	0.68	+
	Thbs2	1.25E-04	0.55	+
	Wasf2	4.43E-03	0.71	-
	Xiap	3.97E-04	0.63	+
PPARγ signaling pathway (72hr)	Acsl1	9.52E-04	0.6	+
	Acox1	8.33E-03	0.77	+
	Adipoq	8.38E-04	0.4	+
	Cd36	1.45E-03	0.48	-
	Fabp5	4.31E-03	0.74	-

Close to eight thousand transcripts were identified to bear m^6^A in mouse brain[16], and we compared the 970 genes differentially regulated by FTO knock-down to this list. Interestingly, 450 out of 970 contain m^6^A sites (S1 Table), again highlighting the important role of demethylase activity of FTO.

## Discussion

In this report, we used mouse 3T3-L1 preadipocyte as a model to study the function of FTO during adipogenesis. FTO is expressed in preadipocyte, and its mRNA level did not show significant changes up to 6 days after differentiation induction. Knock-down of FTO expression with siRNAs in preadipocyte resulted in a decrease of differentiation into mature adipocytes. To confirm the observation with siRNA knock-down, we generated two lines of 3T3-L1 cells stably expressing wild-type FTO, and a FTO mutant (R96Q) lack of demethylase activity. Consistent with the siRNA knock-down experiments, over-expression of ectopic FTO enhanced differentiation, whereas the R96Q mutant failed to do so, and displayed a dominant negative effect. These observations suggest that the demethylase activity of FTO is required during adipogenesis. We further demonstrated that the effect of FTO on differentiation is mediated through PPARγ, a master regulator of adipogenesis. Rosiglitazone, a PPARγ agonist could partially overcome the inhibition effect observed in R96Q expressing cells.

Recent studies suggest m^6^A as the cellular substrate of FTO [15]. Although the presence of m^6^A modifications in RNA was discovered decades ago [45–53], its physiological role remains largely unknown. Transcriptome-wide studies indicate m^6^A modifications are common, highly conserved between mouse and human, and dynamically regulated [16, 17]. In this report, we demonstrated that over-expressing of FTO led to decrease of m^6^A in 3T3-L1 preadipocytes, accompanied by an increase in differentiation level upon adipogenic induction. In contrast, cells expressing R96Q mutant has no effect on m^6^A level. Interestingly, the R96Q mutant displayed a dominant negative effect, possibly through competing for substrate binding with endogenous FTO.

Two research articles were published very recently highlighting one molecular mechanism through which FTO regulated adipogenesis [29, 30]. Runt related transcription factor 1 (RUNX1T1) was previously shown to impact adipogenesis through C/EBPβ [54]. With the application of RNA-seq and m^6^A-seq technology, Zhao et al found that FTO controls the exon splicing of RUNX1T1 by regulating m^6^A levels around splice sites, which led to two isoforms of the protein: the long and the short forms. FTO increases the short form of RUNX1T1 and thus enhances adipocyte differentiation [29]. This observation was also supported in mouse embryonic fibroblasts (MEFs) induced to differentiation into adipocytes [30]. Furthermore, the latter publication demonstrated that FTO exerts its effect by regulating events early in adipogenesis, during the process of mitotic clonal expansion. In a study published in 2013, Tews et al reported that FTO was involved in the white adipose tissue browning, which highlighted the possible role of FTO in regulating energy expenditure [31]. However, knock-down of FTO in human preadipocyte SGBS did not demonstrate an effect on differentiation rate. Our observation that Rosiglitazone could restore differentiation suppression is consistent with the discovery that FTO is upstream of PPARγ, and also explained the discrepancy observed between mouse and human adipocytes, since SGBS was cultured in the presence of rosiglitazone, which probably led to overcoming the differentiation suppression imposed by FTO deficiency.

Using microarray, we explored the transcriptional changes induced by FTO knock-down during 3T3-L1 differentiation. Six hours after induction, an up-regulation of the Wnt pathway was detected. Activation of canonical Wnt signaling by Wnt10b was previously shown to block adipogenesis, possibly through interaction with PPARγ [37, 38, 55]. We detected a mild increase of Wnt10b transcript at 6 hr post induction. At 72 hr, a decrease of PPARγ responsive genes was observed. One possible mechanism of FTO regulating 3T3-L1 differentiation might be via increasing Wnt signaling, and thus suppress PPARγ activity. It would be important for mechanistic understanding to investigate the effect of activation of Wnt pathway on adipogenesis upon ectopic expression of FTO.

Another interesting observation from the microarray data is that many of the transcripts regulated by FTO knock-down contains m^6^A sites (450 out of 970), again highlighting the important role of demethylase activity of FTO. The critical role of demethylase activity has been highlighted in various tissues/cell lines besides adipose/adipocytes. Recently it was demonstrated that FTO could act as link between amino acid sensing and mammalian target of rapamycin (mTOR) signaling, and thus possibly influence body composition through playing a role in cellular nutrient sensing [56]. A demethylase mutant of FTO, R316Q, however, was ineffective in coupling amino acids deprivation to mTOR signaling [56]. In another study, FTO expression is correlated with reduced level of m^6^A in ghrelin mRNA, and postulated to regulate food intake through regulating ghrelin level [57].The more recent studies in adipocytes as well as adipose tissues in FTO-/- or FTO over-expressing mice further demonstrated the critical roles played by the demethylase activity of FTO. Interestingly, over-expression of FTO in MEF cells did not show an impact on m^6^A level [28], suggesting the effect of FTO-dependent demethylation could be tissue-specific and/or temporally regulated. The establishment of 3T3-L1 lines stably expressing FTO and a demethylase mutant will allow further investigation of the demethylation targets of FTO during adipogenesis.

In conclusion, we demonstrated that FTO plays an important role in preadipocyte differentiation, and demethylation modification of m^6^A is required during this process. The effect of FTO is mediated, at least partially through PPARγ. Our study set the basis for further investigation into the molecular mechanisms underlying the involvement of FTO during adipogenesis.

## Supporting Information

S1 Table Genes differentially expressed in siFTO treated 3T3-L1 cell lines. (XLS)
